# Functional Polymeric Materials for Micro- and Nanoplastic Removal from Waters

**DOI:** 10.3390/polym18091081

**Published:** 2026-04-29

**Authors:** Juan Carlos Bravo-Yagüe, Gema Paniagua-González, Rosa María Garcinuño, Asunción García-Mayor, Pilar Fernández-Hernando

**Affiliations:** Department of Analytical Sciences, Faculty of Sciences, National University of Distance Education (UNED), Av. de Esparta s/n, 28232 Madrid, Spain; rmgarcinuno@ccia.uned.es (R.M.G.); mgarcia@ccia.uned.es (A.G.-M.); pfhermando@ccia.uned.es (P.F.-H.)

**Keywords:** microplastics, nanoplastics, functional polymeric materials, adsorption, filtration, coagulation, flocculation, water treatment, polymer nanocomposites

## Abstract

Micro- and nanoplastic pollution poses an emerging challenge for aquatic environments, driving the need for efficient and scalable removal strategies. Functional polymeric materials (FPMs) have emerged as a versatile platform to address this issue, owing to their tunable chemical composition, structural diversity, and ability to promote multiple removal mechanisms, including adsorption, filtration, and coagulation/flocculation. This review provides an overview of recent advances in polymer-based strategies for the removal of micro- and nanoplastics, with emphasis on material design, interaction mechanisms, and process performance. A broad range of materials, including natural hydrogels, polysaccharide aerogels, synthetic polymer composites, magnetic hybrids, and metal–organic frameworks (MOFs)–polymer systems, have demonstrated high removal efficiencies through electrostatic interactions, hydrogen bonding, hydrophobic effects, π–π stacking, and physical entrapment. Removal performance is strongly influenced by surface functionalization, porosity, surface area, and polymer network architecture, enabling targeted design for specific particle types and water matrices. Hybrid and multifunctional materials further enhance capacity and reusability, while natural polymers offer sustainable alternatives. Despite these advances, challenges remain in standardization, scalability, long-term stability, fouling resistance, and economic feasibility under realistic environmental conditions. Future research should focus on sustainable, multi-target, and scalable FPMs, integrating hybrid architectures, stimuli-responsive functionalities, and bioinspired design strategies. Particular attention should be given to mechanistic studies under environmentally relevant conditions and the establishment of structure–property design criteria to enable efficient removal of heterogeneous MPs/NPs mixtures. Overall, functional polymeric materials represent a flexible and high-performance platform for mitigating micro- and nanoplastic contamination, although their successful implementation will depend on bridging the gap between laboratory-scale performance and real-world water treatment applications.

## 1. Introduction

**Figure 1:**
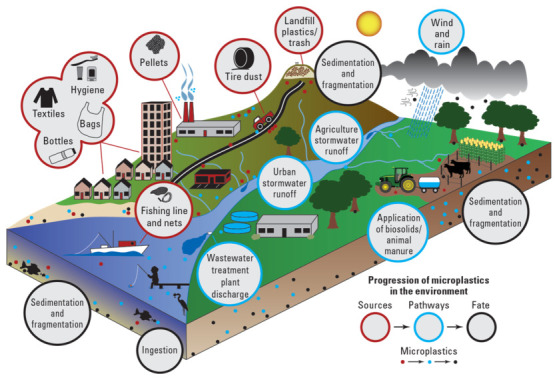
Microplastic sources, pathways, and fate—conceptual diagram. Reprinted from reference [25].

The widespread occurrence of microplastics (MPs) and nanoplastics (NPs) in aquatic environments has emerged as a significant global concern over the past decade [1,2]. These plastic particles, typically defined as smaller than 5 mm in diameter for MPs and below 1 µm for NPs [3,4,5], originate from the fragmentation of larger plastic debris and from primary sources such as synthetic textiles, industrial effluents, and personal care products. Figure 1 shows a conceptual diagram for MP sources, pathways, and fate in aquatic systems.

Due to the high chemical stability and low degradability of most synthetic polymers, MPs and NPs can persist in aquatic ecosystems for extended periods. In addition, their small size and large surface area facilitate dispersion in water and enable them to act as carriers for hydrophobic organic contaminants, heavy metals, and pathogenic microorganisms. Such interactions may promote bioaccumulation, ecotoxicological effects, and potential human health risks through exposure via water and the food chain [6,7,8]. Traditional water and wastewater treatment methods, including conventional filtration, sedimentation, and coagulation, are not fully effective at removing micro- and nanosized pollutants, primarily due to their small size, physicochemical heterogeneity, and interactions with natural organic matter (NOM). Although many wastewater treatment plants report high removal efficiencies for larger MPs, a substantial number of smaller particles, especially fibers and NPs, can persist in treated effluents and be discharged into receiving water bodies [9,10,11].

MPs and NPs are commonly classified according to their polymer type, with polyethylene (PE), polypropylene (PP), polystyrene (PS), and polyvinyl chloride (PVC) among the most frequently reported materials. However, environmental MPs and NPs rarely consist of pure polymers. Instead, they originate from commercial plastic products that contain a wide range of additives, including plasticizers, stabilizers, dyes, and flame retardants. As a result, particles classified under the same polymer type may differ significantly in chemical composition, surface properties, and aging state, which in turn influence their interaction behavior with functional polymeric materials. While most experimental studies rely on model particles with well-defined properties, these complexities highlight the need to interpret laboratory results within the context of real environmental systems.

Therefore, the development of advanced materials capable of efficiently capturing and removing MPs and NPs from water systems has attracted increasing attention. In this context, functional polymeric materials (FPMs) have emerged as a promising strategy to address these challenges. FPMs are polymeric systems, either synthetic or natural, that are specifically engineered or modified to possess additional chemical or physical functions beyond the typical structural and mechanical properties of conventional polymers. These functions arise from the introduction of specific functional groups, tailored macromolecular architectures, or incorporated fillers [12,13]. These tailored chemical and physical properties govern the removal of MPs and NPs through a combination of molecular interactions such as electrostatic attraction, hydrophobic interactions, and π–π stacking, and retention mechanisms, such as physical entrapment within the porous structure and size exclusion. The overall efficiency depends on both the material design and the physicochemical properties of the plastic particles.

FPMs offer several advantages over conventional water treatment approaches. They can provide high surface area, adjustable porosity, and enhanced affinity for MPs and NPs, as well as the potential for regeneration and reuse, making them particularly suitable for water treatment applications. Furthermore, these materials can be integrated into hybrid systems, such as polymer–membrane composites or polymer–nanomaterial hybrids, thereby combining multiple removal mechanisms for enhanced efficiency. Recent studies have demonstrated successful applications of a variety of FPMs, including natural polymer-based hydrogels, magnetic polymer nanocomposites, and functionalized polymeric membranes, for the elimination of MPs and NPs from rivers, lakes, and wastewater effluents, highlighting their potential for large-scale environmental remediation [14,15,16].

Despite these advantages, the practical application of FPMs is still limited by several challenges, including fouling susceptibility, limited scalability, and long-term stability, which are further discussed in the Critical Evaluation and Research Gaps Section.

In this context, in recent years, the scientific literature has seen a proliferation of review articles addressing the removal of MPs and NPs from water systems. Several comprehensive reviews have summarized the wide range of separation and treatment technologies available, from physical filtration and coagulation-flocculation to degradation processes. For example, Chen et al. [17] provide a broad overview of separation and degradation methods for MPs/NPs in urban waters, covering adsorption, flotation, filtration, magnetic separation, and more traditional approaches. Reviews also exist that focus on specific techniques. Tang et al. [18] have systematically reviewed coagulation strategies for the capture of MPs, emphasizing mechanisms like charge neutralization and sweep flocculation. Acarer [19] reviewed membrane filtration for MPs in water and wastewater.

However, while some reviews focus on specific methods, for instance, adsorption by general adsorbents without polymeric specialization [20,21], or nanomaterials in wastewater treatment contexts [22,23], none to date has synthesized the rapidly growing body of work on FPMs as a unified class of solutions for MP and NP removal. Furthermore, recent bibliometric analyses highlight that although adsorption is recognized as a promising mechanism, the detailed roles of polymer structure, surface functionalization, and hybrid material design have not been fully integrated and critically reviewed within a single framework [24].

Despite this richness of literature and the growing number of review articles, information on FPMs remains dispersed across studies that treat them as one among many adsorbent types, membrane components, or hybrid systems. No review to date has systematically consolidated the diverse classes of FPMs into a unified framework that allows researchers to compare material performance and identify promising directions for engineering next-generation polymeric solutions.

Therefore, this review aims to fill this gap by providing a comprehensive and coherent analysis of FPMs for the removal of MPs and NPs from aqueous environments, enabling comparison of material performance and identification of promising directions for next-generation polymeric solutions.

## 2. Classification of Functional Polymeric Materials

FPMs for water pollutants removal encompass a wide variety of architectures and chemistries designed to promote interactions with target compounds in aqueous environments. Their classification is primarily based on structural architecture (e.g., porous networks, membranes), material composition (synthetic versus natural), and functional design. These parameters collectively govern key performance characteristics, including accessibility of active sites, affinity toward target compounds, mechanical stability, and compatibility with water treatment operations.

**Figure 2:**
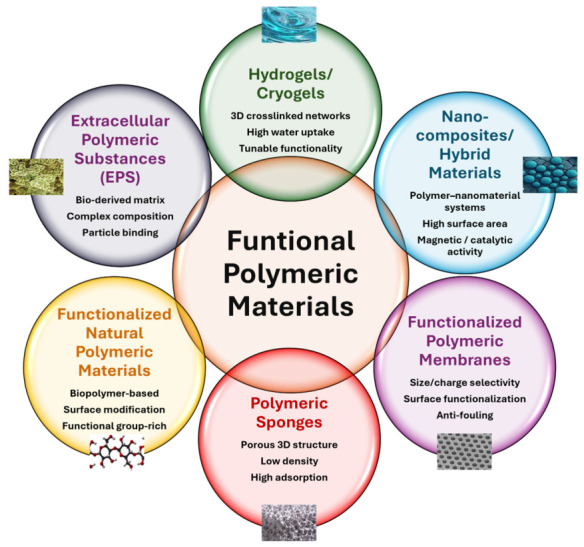
Classification of functional polymeric materials (FPMs) for microplastic (MP) and nanoplastic (NP) removal.

Based on these considerations, FPMs can be broadly classified into: (i) hydrogels and cryogels, (ii) polymeric nanocomposites and hybrid materials, (iii) functionalized polymeric membranes, (iv) polymeric sponges, (v) functionalized natural polymeric materials, and (vi) extracellular polymeric substances (EPS). A schematic representation of this classification is provided in Figure 2.

It is important to note that these categories are not mutually exclusive, as many FPMs integrate multiple structural and functional features within a single system. Nevertheless, a structured classification provides a useful framework for rationalizing design strategies and enabling meaningful comparisons among different material platforms.

### 2.1. Hydrogels and Cryogels

Hydrogels are three-dimensional cross-linked polymer networks with high water uptake capacity, controllable pore size, and adjustable surface chemistry, making them highly versatile materials for water purification applications. Their network architecture, which can be tailored via chemical or physical crosslinking, governs water retention, mechanical stability, and the accessibility of functional groups for interaction with dissolved species and particulate matter.

**Figure 3:**
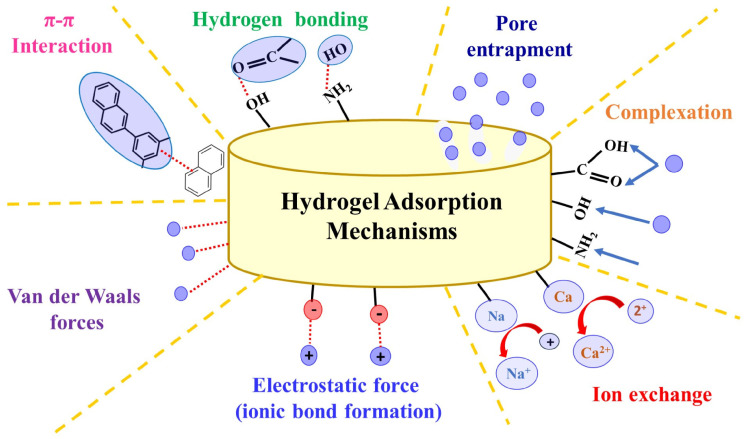
Adsorption mechanisms of hydrogels for the removal of contaminants. Reprinted from reference [36].

The distinctive physicochemical properties of hydrogels, including high porosity and engineered surface functionality, are important for their performance in sorption and transport processes in aqueous systems [26]. A schematic representation of different adsorption mechanisms of hydrogels for the removal of contaminants is shown in Figure 3. A wide range of synthesis and functionalization strategies, including conventional polymerization, crosslinking, and incorporation of nanomaterials or composite phases, enables precise control over network density, swelling behavior, and contaminant affinity [27,28]. Preparation methods have been extensively reviewed, highlighting how polymer backbone selection, crosslinking mechanisms, and inclusion of natural or synthetic additives influence structural and functional properties [29]. In this context, hydrogel-based systems have been widely explored for the capture of MPs and NPs, particularly PS particles, through π–π stacking and electrostatic interactions [30].

Natural polymer-based hydrogels, particularly those derived from polysaccharides and other biopolymers, have attracted significant attention due to their biodegradability, biocompatibility, and functional versatility. These systems can be chemically or physically modified to tune mechanical properties, porosity, and sorption capacity, enabling interaction with a broad range of aqueous contaminants [31]. These materials have shown particular promise for the removal of MPs and NPs due to their high density of functional groups, which promote the binding and retention of polymeric particles in water [32].

Cryogels, a subclass of hydrogels formed via freeze–thaw or cryogelation processes, exhibit interconnected macroporous architectures that facilitate rapid mass transport. This structural feature enables fast fluid penetration and efficient entrapment of suspended particulate matter due to their large pore channels. Recent studies in environmental remediation and wastewater treatment have demonstrated how cryogel network design and functionalization can be optimized to enhance pollutant uptake and reusability [33,34]. In the case of MPs/NPs, cryogels have demonstrated effective removal not only through physical entrapment within their macroporous networks, but also via aggregation-mediated mechanisms that enhance subsequent filtration [35].

### 2.2. Polymeric Nanocomposites and Hybrid Materials

Polymeric nanocomposites consist of a polymer matrix integrated with nanoscale fillers such as metal or metal oxide nanoparticles, graphene, carbon nanotubes, layered silicates, or metal–organic frameworks (MOFs). This integration enhances surface area, mechanical strength, and functional diversity compared with pristine polymers.

The incorporation of nanomaterials improves intrinsic polymer properties such as hydrophilicity, porosity, and structural stability, while simultaneously introducing additional active sites for adsorption, catalysis, or charge-driven interactions [37]. The synthesis of polymer nanocomposites involves diverse approaches, including in situ polymerization, blending, electrospinning, phase inversion, and interfacial assembly, which allow precise control over filler dispersion and polymer–nanofiller interactions [38,39]. Uniform nanofiller distribution is essential for maximizing accessible surface area and preventing aggregation, which could otherwise compromise performance and mechanical integrity. In addition, polymer matrices can be chemically modified to improve compatibility with nanofillers, thereby tuning swelling behavior, permeability, and antifouling properties.

Hybrid materials extend this concept by integrating multiple functional components capable of synergistic processes such as adsorption, photocatalysis, enzyme catalysis, or charge transfer. Interface engineering strongly influences reactivity and long-term stability [27]. Examples include layered double hydroxides, MOFs, carbon-based composites, and biohybrid systems, which exhibit enhanced adsorption kinetics and multifunctional behavior under aqueous conditions [40]. In the context of MP/NP removal, polymer-based hybrid systems incorporating nanofillers or MOFs have demonstrated enhanced performance, driven by synergistic adsorption mechanisms—including electrostatic interactions, hydrophobic forces, hydrogen bonding, and van der Waals interactions—that promote particle binding and retention [41]. Despite their promising performance, challenges remain regarding scalability, stability over extended periods, formation of potential by-products, and the ability to reproduce results in real water matrices. Recent studies emphasize the importance of assessing the life cycle, validating processes at the reactor scale, and using energy-normalized metrics to bridge the gap between laboratory experiments and practical deployment [42].

### 2.3. Functionalized Polymeric Membranes

Polymeric membranes are continuous polymeric structures with controlled pore-size distributions, widely used in water and wastewater treatment. Their ability to separate species based on size, charge, or chemical affinity makes them essential in processes such as microfiltration, ultrafiltration, nanofiltration, and reverse osmosis. Common base polymers include polyethersulfone (PES), polyvinylidene fluoride (PVDF), and polysulfone (PSF), selected for their mechanical strength and chemical stability.

Functionalization strategies are key for improving membrane performance and include chemical grafting, surface coating, blending, and layer-by-layer assembly. These approaches enable tuning of hydrophilicity, surface charge, porosity, and antifouling properties [43]. The incorporation of nanomaterials such as graphene oxide (GO), carbon nanotubes (CNTs), TiO_2_, ZnO, and MOFs, as well as biopolymers like chitosan (CS) and cellulose, further enhances membrane functionality by increasing active sites and improving mechanical stability [27]. A comprehensive discussion of these aspects is provided by [44]. These membranes have also been widely investigated for the removal of MPs and NPs from aqueous systems, where separation is primarily governed by size exclusion, complemented by surface-mediated interactions depending on particle size and membrane chemistry. In particular, functionalized membranes incorporating nanomaterials or biopolymers have shown enhanced performance in the removal of MPs and NPs such as PS and PE, due to improved surface affinity and reduced fouling tendency [45,46].

It is important to distinguish the role of membrane functionalization in MP/NP removal. While size exclusion and cake layer formation dominate the retention of MPs, membrane functionalization primarily enhances surface properties such as charge, hydrophilicity, and antifouling behavior, which can improve the interaction with submicron particles. In this context, NPs may additionally be influenced by interfacial interactions at the membrane surface, contributing to their partial retention.

**Figure 4:**
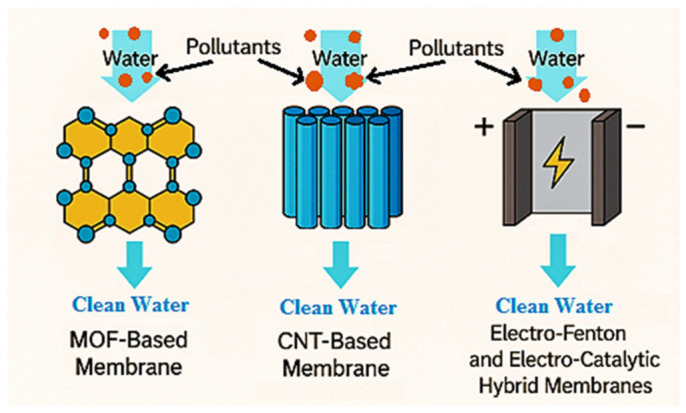
Schematic representation of carbon nanotubes, MOFs, and electro-Fenton-integrated advanced functional membranes (AFMs). Reprinted from reference [47].

Advanced functional membranes (AFMs) integrating MOFs or carbon-based materials can enhance MP and NP removal by combining size exclusion with interfacial interactions and, in some cases, catalytic functions. Hybrid membrane systems further expand functionality through photocatalytic or electrochemical processes. Figure 4 shows a schematic representation of carbon nanotubes, MOFs, and electro-Fenton-integrated AFMs [47]. These systems have demonstrated high efficiency in the retention of MPs/NPs particles in water, where physical sieving and cake layer formation dominate for MPs, while interfacial interactions may contribute to nanoparticle retention [48].

However, challenges remain in scaling up these systems, including long-term stability, cost of fabrication, reproducibility, and the environmental impact of modifying agents. Future research focuses on sustainable fabrication methods, antifouling strategies, and integration with complementary treatment processes. This is particularly relevant for the treatment of real waters containing complex mixtures of MPs, NPs, and NOM.

### 2.4. Polymeric Sponges

Polymeric sponges are highly porous three-dimensional materials characterized by interconnected macroporous networks that enable efficient physical entrapment of particulate matter. Their high porosity and tunable surface properties make them attractive for environmental remediation and water treatment applications [49].

**Figure 5:**
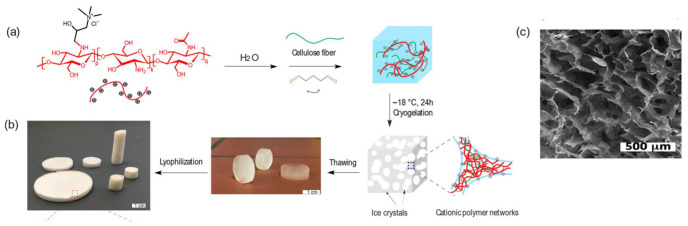
Schematic illustration of (**a**) Preparation of polycationic sponge materials by cryo-polymerization; (**b**) image of prepared cryogel sponges in the swollen and dry state; (**c**) scanning electron microscopy (SEM) image of cryogel sponges. Reprinted from reference [50].

Synthesis approaches include templating, freeze-drying, and cryogelation, often combined with chemical functionalization to introduce functional groups such as amines, carboxylates, or quaternary ammonium moieties. These modifications tailor surface charge, wettability, and interfacial affinity, thereby governing adsorption efficiency, particle capture, and flocculation behavior at the solid–liquid interface. For example, cationic modifications can enhance particle binding, while composite structures improve mechanical stability during repeated use [50]. In the context of MP/NP removal, such functionalized sponges have shown enhanced ability to capture polymeric particles such as PS, primarily through a combination of electrostatic attraction and physical entrapment within the porous network [51]. Figure 5 shows the scheme of preparation of a polycationic sponge material made from CS derivatives and cellulose fibers.

From a mechanistic perspective, contaminant removal by polymeric sponges is governed by coupled transport and interfacial processes within their hierarchical porous networks. The interconnected structure enables efficient fluid uptake and promotes transport of contaminants into the internal pore system, where retention occurs through a combination of physical entrapment, size exclusion, and interfacial interactions with functionalized pore surfaces. Surface chemistry and wettability further influence the affinity for different pollutants, while the elasticity of the sponge allows structural deformation that improves contact between contaminants and active sites. Overall, the interplay between pore architecture, surface properties, and mechanical response governs the overall capture efficiency and reusability of these materials in aqueous environments.

Recent studies have demonstrated the effectiveness of polymeric sponges in oil–water separation, adsorption, and interfacial processes. CS–graphene composite sponges exhibit high oil uptake capacities due to their controlled porosity and surface wettability [52]. Although many studies have demonstrated design principles for oil–water separation systems, similar principles are directly transferable to the capture of MP particles in aqueous environments. Polydimethylsiloxane (PDMS)-based sponges have also been developed for interfacial solar steam generation, enabling continuous desalination and pollutant recovery while providing self-regeneration and reversible wettability control [53]. In particular, such interfacial systems provide a conceptual basis for extending sponge-based materials toward MP/NP removal applications. Further details on the application of polymeric sponges for MP/NP removal and the associated interaction mechanisms are discussed in Section 3.

### 2.5. Functionalized Natural Polymeric Materials

Functionalized natural polymers, including cellulose, CS, alginate, starch, and gelatin, represent a sustainable class of materials for water purification due to their renewable origin, biodegradability, and rich surface chemistry. These polymers contain abundant functional groups such as hydroxyl, amino, and carboxyl groups, enabling diverse interactions with contaminants including ion exchange, hydrogen bonding, and electrostatic attraction.

However, native biopolymers often suffer from limited surface area and poor mechanical stability. Therefore, functionalization strategies such as oxidation, grafting, crosslinking, blending with nanomaterials, or incorporation of magnetic particles are widely employed to enhance adsorption capacity and reusability [54,55].

Chitosan, in particular, has been extensively studied for its biocompatibility and functional versatility, although its practical applications are limited by its low solubility and mechanical weakness. Chemical modification and composite formation significantly improve its performance for contaminant removal [56]. Similarly, cellulose-based materials can be functionalized through surface activation and composite formation to yield bioadsorbents with high affinity for a variety of pollutants. Polysaccharide-based materials derived from cellulose, alginate, and related biopolymers have been widely investigated, showing excellent adsorption capacity for MPs and NPs [57], in addition to other aqueous contaminants such as heavy metals and organic contaminants. These systems demonstrate promising performance in terms of particle capture, reusability, and facile recovery, owing to their hydrophilicity and abundant functional groups [58].

### 2.6. Extracellular Polymeric Substances (EPS)

Porous extracellular polymeric substances (EPS) are biopolymeric matrices secreted by microorganisms such as bacteria, microalgae, and fungi. They are major structural components of biofilms, flocs, and granular sludge and consist mainly of polysaccharides, proteins, lipids, nucleic acids, and humic-like substances.

EPS form hydrated three-dimensional networks enriched in functional groups such as hydroxyl, carboxyl, amino, sulfhydryl, and phosphate groups, which enable strong interactions with dissolved and particulate species through electrostatic attraction, hydrogen bonding, and complexation [59]. Within this physicochemical framework, EPS act as reactive matrices capable of interacting with MPs and NPs, leading to their aggregation, immobilization, or incorporation into bioaggregates. These processes emerge from coupled effects of EPS structural properties and solution chemistry, which together govern particle adhesion and stability within the EPS matrix. In this context, EPS have been shown to facilitate the aggregation and immobilization of MPs/NPs through a combination of electrostatic interactions and polymer bridging effects [60]. A key parameter controlling these interactions is the protein-to-polysaccharide ratio, which influences polymer conformation, surface charge distribution, and the availability of binding sites. This compositional balance determines whether interactions with MPs/NPs are dominated by electrostatic attraction, steric stabilization, or bridging flocculation. In general, protein-rich EPS enhance hydrophobic and electrostatic interactions with MPs/NPs, promoting compact aggregation structures, whereas polysaccharide-rich matrices tend to favor steric stabilization or, under appropriate ionic strength, bridging-mediated aggregation. These mechanisms are particularly relevant for the incorporation of MPs and NPs into bioaggregates in wastewater treatment systems.

**Figure 6:**
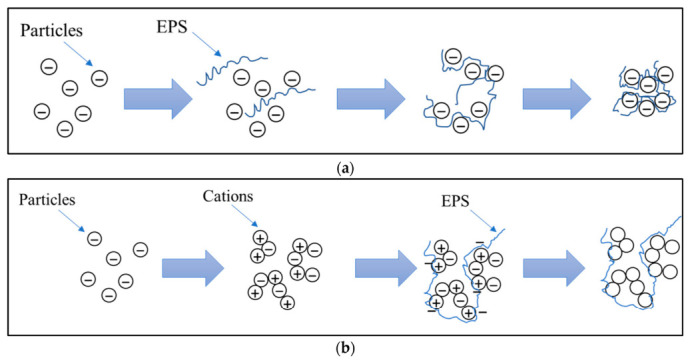
Mechanism of flocculation in wastewater treatment promoted by EPSs via (**a**) bridging creation and (**b**) patch flocculation. Reprinted from reference [62].

In biological wastewater treatment systems, these compositional and physicochemical factors further govern EPS-mediated bioaggregation and flocculation by regulating interactions between microbial cells, suspended particles, and MPs/NPs through chain entanglement and electrostatic forces. Multivalent cations such as Ca^2+^ and Mg^2+^ further enhance these processes by forming ionic bridges between negatively charged functional groups, strengthening network cohesion, and improving floc stability. These coupled mechanisms regulate the incorporation and retention of MPs/NPs within biological flocs [61,62]. This process plays a central role in the removal of MPs/NPs in wastewater treatment, where EPS-mediated flocculation promotes particle capture and sedimentation. Figure 6 shows a schematic representation of the mechanism of flocculation in wastewater treatment promoted by EPSs.

Beyond structural roles, EPS also exhibit intrinsic adsorption capacity for MPs such as PP and PET [63], in addition to other aqueous contaminants. Their binding behavior depends on composition and environmental conditions, such as pH and ionic strength, which affect charge density, polymer configuration, and cation availability, thereby modulating the balance between aggregation and stabilization processes. EPS formation and composition are influenced by microbial diversity and operational conditions such as nutrient availability, temperature, pH, and the presence of toxic substances. Different microbial communities produce EPS with varying proportions of polysaccharides, proteins, and humic-like substances, which, in turn, affect functional properties relevant to aggregation and contaminant interactions. These properties are therefore directly relevant to the fate and removal of MPs/NPs in biological water treatment systems. For example, tightly bound EPS fractions have been associated with stronger flocculating activity than loosely bound fractions due to greater functional group density and chain entanglement [64].

## 3. Mechanisms and Processes for Micro- and Nanoplastic Removal

MP and NP removal in aqueous environments can occur through a variety of interaction mechanisms and process-driven strategies. This section focuses on representative approaches employing FPMs, including adsorption, physical retention (filtration), and coagulation/flocculation.

MPs and NPs interact with FPMs via combinations of physical, chemical, and surface-specific mechanisms, which together determine removal efficiency and overall separation performance in practical applications. These interactions also influence the stability, reusability, and scalability of the treatment process. In most cases, a single FPM does not rely on one mechanism alone; rather, adsorption, filtration, and aggregation operate synergistically, with their relative contributions shaped by particle and polymer properties, as well as environmental conditions such as pH, ionic strength, temperature, and water matrix composition.

**Figure 7:**
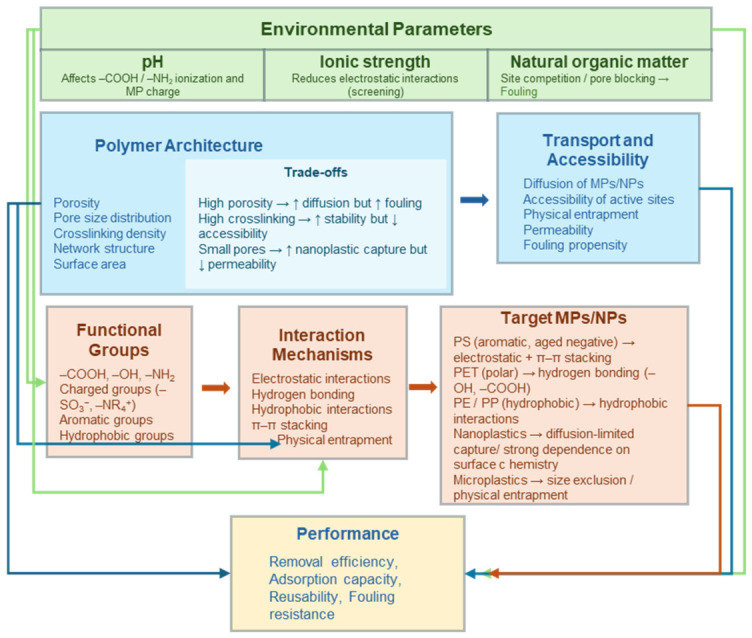
Conceptual framework linking functional polymer properties with interaction mechanisms and removal performance toward micro- and nanoplastics (MPs/NPs), highlighting how environmental parameters and design trade-offs influence overall efficiency.

To provide a unifying perspective across these processes, a conceptual framework is presented in Figure 7, establishing explicit relationships between functional groups, polymer architecture, interaction mechanisms, and target MP/NP characteristics, within the context of relevant environmental parameters. Specifically, functional groups (e.g., -NH_2_, -COOH, aromatic groups) are associated with dominant interaction mechanisms such as electrostatic attraction, hydrogen bonding, hydrophobic interactions, and π–π stacking, which, in turn, determine affinity for different types of MPs/NPs depending on their surface chemistry and size.

In parallel, polymer architecture, including porosity, pore size distribution, and crosslinking density, is linked to transport phenomena such as diffusion, accessibility of active sites, and physical entrapment, which are particularly relevant in filtration and hydrogel-based systems. Environmental factors such as pH, ionic strength, and NOM further modulate these interactions by altering surface charge, screening electrostatic forces, and competing for active sites.

This integrative framework provides a structure–property–mechanism–performance relationship that serves as a basis for interpreting the different removal strategies discussed below.

The following sections describe adsorption, filtration, and coagulation/flocculation processes, emphasizing the key factors governing their performance and how they relate to the framework presented in Figure 7.

### 3.1. Adsorption

Adsorption is one of the most widely applied strategies for the removal of MPs and NPs from aqueous environments using FPMs. In this process, plastic particles (adsorbates) interact with the surface or internal structure of an adsorbent through a combination of physicochemical forces, including electrostatic attraction, hydrophobic effects, π–π stacking, hydrogen bonding, coordination interactions, and, in some cases, physical entrapment within porous structures [65,66]. The overall efficiency of adsorption is determined by both the properties of the adsorbent and the characteristics of the plastic particles, including size, surface charge, chemical composition, and degree of environmental aging.

From a mechanistic perspective, adsorption generally proceeds through a sequence of mass-transfer steps. Particles first migrate from the bulk solution toward the adsorbent surface through advection, dispersion, or molecular diffusion. Once near the surface, they cross the boundary layer at the solid–liquid interface and may subsequently diffuse into the internal pore structure before interacting with active adsorption sites. These sequential steps, commonly described as external diffusion, internal diffusion, and surface adsorption, are fundamental for understanding and optimizing material performance.

Adsorption can be broadly classified into physisorption and chemisorption, representing a continuum of interaction strengths rather than strictly separated regimes. Physisorption is governed by relatively weak forces such as van der Waals interactions, hydrophobic effects, hydrogen bonding, and π–π stacking and is generally reversible. Chemisorption involves stronger and more specific interactions, including covalent or coordination bonding, leading to higher binding energies and often irreversible attachment [67].

In FPM-based systems, surface chemistry and functionalization play a key role in adsorption efficiency. Functional groups such as amine, hydroxyl, carboxyl, or phenolic groups can promote electrostatic attraction or hydrogen bonding with plastic particles. Hydrophobic modifications favor the removal of nonpolar polymers such as PE and PP, while aromatic structures facilitate π–π interactions with PS [30]. High surface area, hierarchical porosity, and interconnected networks, as found in hydrogels, aerogels, sponges, and nanofibrous membranes, provide abundant accessible binding sites and enable partial pore entrapment [68].

Environmental parameters such as pH, ionic strength, and temperature further modulate adsorption by affecting both the surface chemistry of the adsorbent and the properties of the plastic particles. In addition, NOM and dissolved ions may compete for adsorption sites, highlighting the importance of evaluating these materials under realistic water conditions [69,70].

Several studies have demonstrated the effectiveness of polymeric materials for the adsorption of MPs/NPs in aquatic systems. Natural polymeric systems, particularly polysaccharide-derived hydrogels and aerogels, have shown outstanding performance. For instance, a chitin-cationic lignin composite hydrogel exhibited an exceptionally high maximum adsorption capacity of 1790.8 mg·g^−1^ for PS NPs under neutral pH conditions, maintaining over 93% efficiency after several reuse cycles due to synergistic electrostatic interactions and π–π stacking [32]. Similarly, sodium alginate hydrogels modified with polydopamine (PDA) achieved removal efficiencies of approximately 99.6% with maximum adsorption capacities of 127.98 mg·g^−1^ for amino-functionalized-PS (PS–NH_2_) and 154.57 mg·g^−1^ for carboxylated-PS (PS-COOH), highlighting the role of multiple concurrent interaction mechanisms [30]. An interpenetrating polymer network hydrogel composed of poly(N-isopropylacrylamide) (PNIPAM) and sodium carboxymethyl cellulose (NaCMC) achieved up to 99.83% removal efficiency and 199.64 mg·g^−1^ adsorption capacity for positively charged amine-modified PS (<100 nm) in both seawater and tap water, combining chemisorption, electrostatic interactions, hydrogen bonding, π–π stacking, and pore-filling effects [71].

**Figure 8:**
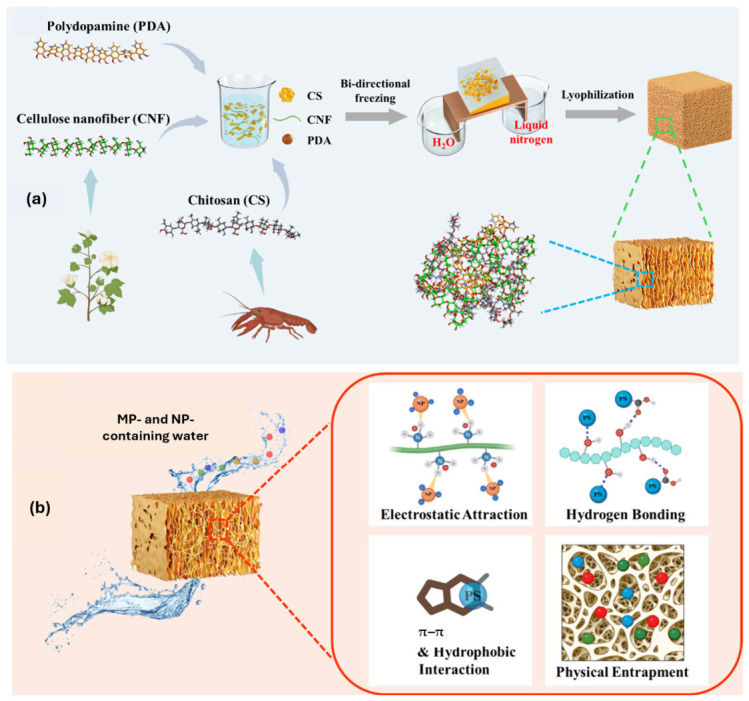
Schematic illustration of (**a**) fabrication procedures for bidirectional chitosan–cellulose nanofiber–polydopamine aerogel, and (**b**) micro- and nanoplastic removal by an adsorptive filtration system through multiple intermolecular interactions. Reprinted from reference [72].

Beyond hydrogels, polysaccharide-based aerogels with bidirectional porous structures composed of CS, cellulose nanofibers, and PDA were synthesized (Figure 8). These hydrogels demonstrated adsorption capacities exceeding 300 mg·g^−1^ for a wide range of plastics (PS-COOH, PS, poly(methyl methacrylate) (PMMA), PE, and PP), achieving removal efficiencies above 96% in continuous-flow systems within 20 min [72].

Similarly, bidirectional cationic modified nanocellulose–(GO) aerogels were synthesized and used for the adsorption of PS microparticles in water, obtaining high efficiencies even after 20 adsorption cycles [73]. Zhuang et al. [74] prepared a cellulose nanofiber aerogel modified with 2, 3-epoxypropyl trimethyl ammonium chloride for the adsorption of PS nanoparticles in water, achieving a good adsorption capacity of 146.38 mg·g^−1^. The same author synthesized nanocellulose-based aerogels functionalized with polyethyleneimine (PEI) that exhibited rapid adsorption kinetics and capacities up to 117 mg·g^−1^ for PS, driven by electrostatic interactions and physical confinement within porous networks [75].

In addition to these polysaccharide-based aerogels, other biodegradable polymers (e.g., polylactic acid and polyhydroxybutyrate) have been considered as sustainable building blocks for the development of porous and aerogel-like structures [76,77]; however, their application in MP/NP removal remains largely unexplored and represents a potential research direction rather than an established strategy.

**Figure 9:**
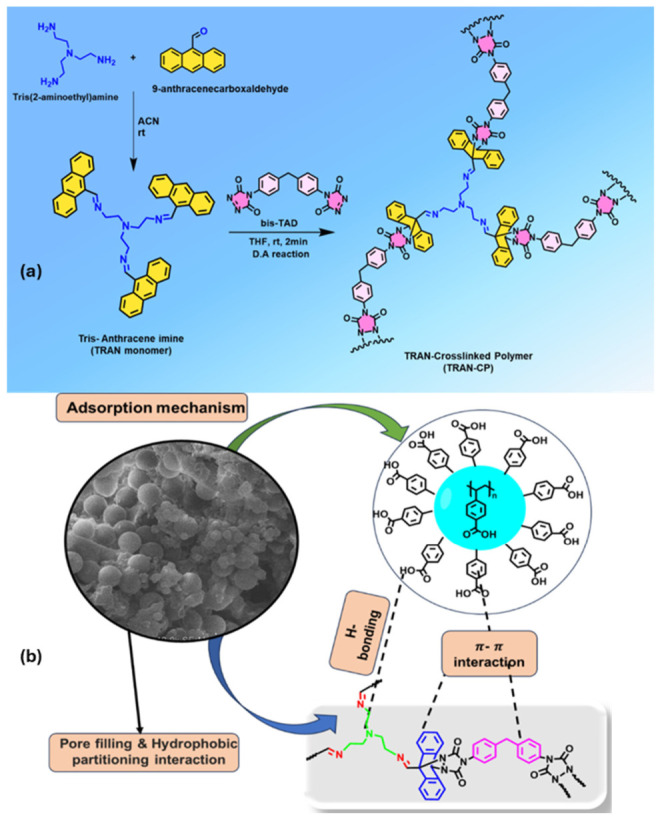
Schematic illustration of (**a**) synthesis of TRAN-CP by ultrafast Diels–Alder click reaction; (**b**) possible mechanism of MP binding onto the TRAN-CP. Adapted from [78]. Copyright © 2026 American Chemical Society.

Synthetic polymer networks further expand adsorption capabilities through tunable chemistry and structural design. For example, a multifunctional hydrophobic porous polymer was synthesized via Diels–Alder click reaction between a trifunctional anthracene monomer and a bis-triazolinedione monomer. The obtained polymer (TRAN-CP) achieved ~190 mg·g^−1^ capacity and ~99% removal efficiency, maintaining high performance in real waters and under column conditions, as well as over multiple regeneration cycles. Figure 9 illustrates the synthesis of TRAN-CP by ultrafast Diels–Alder click reaction and the possible mechanism of MP binding [78].

Hybrid polymeric systems have emerged as a particularly versatile class of adsorbents by combining organic polymers with carbonaceous materials or layered inorganic structures. Polymer-carbon and polymer-layered double hydroxides (LDH) composites, such as chitin–GO sponges and cellulose/MgAl LDH-based materials, have demonstrated effective adsorption of both PS micro- and nanoparticles through multiple interaction pathways, including electrostatic attraction, hydrogen bonding, and π–π interactions [51,57,79,80].

A distinct subgroup of hybrid materials includes magnetically responsive polymeric adsorbents that enable both efficient adsorption and facile recovery. These systems incorporate magnetic nanoparticles (e.g., Fe_3_O_4_) into polymer matrices, allowing rapid separation under external magnetic fields. Magnetic biochar-based systems and upcycled cigarette butt-derived composites demonstrated high removal efficiencies in both model and real water matrices, highlighting the potential of low-cost and sustainable adsorbents [81,82,83]. The multifunctional composite Fe_3_O_4_/poly(sodium p-styrenesulfonate) (PSS)/zeolitic imidazolate framework (ZIF), Fe_3_O_4_/PSS/ZIF-67, further exemplifies this approach, achieving adsorption capacities of 2420–2897 mg·g^−1^ for multiple plastic types (PE, polyethylene terephthalate (PET), PP, PS, etc.) while maintaining a good reusability [84].

Finally, the integration of MOFs with polymeric matrices has emerged as an effective strategy to further enhance adsorption performance. MOF-polymer composites combine the high surface area and tunable porosity of MOFs with the chemical versatility and mechanical stability of polymers. For instance, ZIF-8-based wood aerogels achieved removal efficiencies above 85–91% for poly(1,1-difluoroethylene) and PS nanoparticles through combined electrostatic and hydrogen-bonding interactions [85]. Similarly, PDA-coated diatomite/ZIF-8 composites demonstrated rapid adsorption of PS nanospheres (>91% within 5 min) with good reusability [86]. Other systems, such as ZIF-8 grown on sodium alginate frameworks or mesoporous UiO-66-NH_2_/P123 composites, achieved high adsorption capacities (up to 594 and 524 mg·g^−1^, respectively) for diverse MPs and NPs by combining hierarchical porosity with functional surface groups [41,87].

While intermolecular interactions such as electrostatic attraction, hydrogen bonding, hydrophobic effects, and π–π stacking govern particle binding at the molecular level, the overall performance of an adsorbent is ultimately determined by its surface chemistry, porosity, and structural design. The studies summarized in Table 1 illustrate how these parameters translate into measurable adsorption capacities and removal efficiencies across different particle types and sizes. In this context, oleogel-type organogels, based on hydrophobic networks, may also represent an emerging alternative for MP/NP capture; however, their application in aqueous systems remains largely unexplored compared to the systems discussed above.

### 3.2. Filtration

Filtration represents a primary physical retention strategy for the removal of MPs and NPs from aqueous environments. Filtration processes can be broadly classified into microfiltration (MF), ultrafiltration (UF), and nanofiltration (NF), which differ primarily in pore size and therefore in their dominant retention mechanisms. MF membranes typically exhibit pore sizes in the range of ~0.1–10 µm, UF membranes correspond to effective pore sizes of ~0.01–0.1 µm, and NF membranes present characteristic pore sizes on the order of ~0.001–0.01 µm [88], although these values are strongly dependent on material composition and fabrication method. In addition to pore size, membrane performance is often characterized by the molecular weight cut-off (MWCO), which provides a practical indication of the size range of species retained. MWCO is more commonly applied to UF and NF membranes, typically ranging from 1–500 kDa and below 1 kDa, respectively [89], although these ranges vary depending on membrane material and structure. While MWCO is primarily defined for dissolved species, it provides a useful comparative framework to assess the retention of NPs, particularly in UF and NF systems where size exclusion alone is insufficient.

In contrast, tighter membrane processes such as nanofiltration (NF) and reverse osmosis (RO) may offer higher retention of nanoscale plastics; however, their higher energy consumption, lower permeability, and susceptibility to fouling limit their practical implementation for MP/NP removal. As a result, current research has focused primarily on enhancing MF and UF systems through material functionalization rather than relying on tighter membranes.

Within this framework, MPs (commonly defined in the range of 1 µm to 5 mm) are primarily retained by size exclusion in MF systems; however, particles at the lower end of this size range may not be completely rejected depending on membrane pore size distribution and operational conditions and can also be partially retained in UF processes. In contrast, NPs (<1 µm), particularly those approaching or below UF pore dimensions, are not efficiently removed by size exclusion alone. Their retention increasingly depends on additional mechanisms such as electrostatic interactions, hydrophobic effects, hydrogen bonding, and adsorption phenomena, which become especially relevant in UF and NF regimes [90].

The performance of polymer-based filtration systems is strongly influenced by particle properties (e.g., size, shape, density, and surface chemistry) and membrane characteristics, including pore size distribution, porosity, surface charge, and hydrophilicity/hydrophobicity [91]. It is also strongly dependent on feed water characteristics, including suspended solids, NOM, and salinity, which can significantly influence fouling propensity and overall removal efficiency. In realistic water matrices, NOM and colloidal species may compete with MPs and NPs for adsorption sites or promote the formation of complex fouling structures, including cake layers and pore blockage, thereby reducing permeability and altering separation efficiency over time.

Membranes with pore sizes below typical MP dimensions can achieve high rejection efficiencies, although complete removal is not always guaranteed due to pore-size heterogeneity and operational factors. In contrast, submicron plastics may pass through size-exclusion-based barriers unless additional physicochemical interactions are promoted, for example, through surface functionalization, coatings, or embedded active layers. Accordingly, while pressure-driven membrane processes relying predominantly on size exclusion are generally effective for MPs removal, NPs retention is governed by a combination of partial size restriction and surface-mediated interactions.

Fouling, arising from particle deposition, pore blocking, and cake layer formation, represents one of the main operational limitations of membrane processes, leading to flux decline and reduced long-term performance. This phenomenon is strongly affected by feed water composition and operational conditions and is widely reported in the reviewed studies as a key factor limiting membrane efficiency and durability [92]. While many studies report high removal efficiencies under controlled laboratory conditions, these effects highlight the importance of evaluating membrane performance under more realistic environmental water matrices.

To address these limitations, FPMs have emerged as a promising strategy to improve membrane performance, particularly for submicron NPs by enabling controlled pore architecture, tailored surface charge, and tunable hydrophilic or hydrophobic domains. FPMs can enhance particle capture, mitigate fouling, and improve operational stability [93,94]. While chemical modifications, whether surface-based or involving functional groups, may offer additional advantages in terms of fouling reduction or electrostatic attraction of very small particles, they are not strictly necessary to achieve high MP retention with ultrafiltration and microfiltration membranes; consequently, research specifically focusing on chemically functionalized membranes for MP removal remains limited.

**Figure 10:**
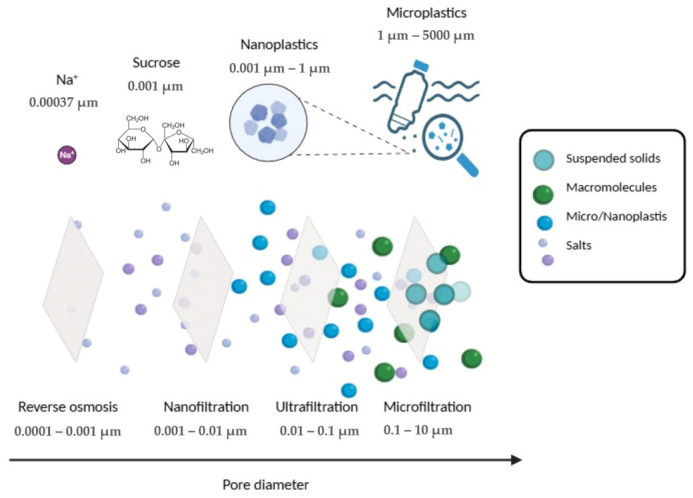
Pressure-driven membrane processes and a range of membrane pore diameters for the removal of MPs and NPs. Reprinted from reference [88].

Pressure-driven membrane processes in which exclusion occurs by size are represented in Figure 10.

Beyond geometric exclusion, surface interactions between plastics and filtration media play a crucial role in both removal efficiency and fouling behavior. Electrostatic attraction or repulsion, hydrophobic affinity, and surface roughness influence particle deposition and retention. However, these same interactions can also lead to fouling through cake layer formation or pore blockage, which increases hydraulic resistance over time. To mitigate fouling while maintaining high retention efficiency, strategies such as hydrophilic coatings, charged polymer layers, or nanocomposite functionalization are commonly employed [95,96].

Recent studies highlight the effectiveness of functional polymer-based filtration systems for MP/NP removal, emphasizing the combined role of material design, surface chemistry, and operating conditions.

Natural membranes have emerged as versatile platforms that combine size exclusion with surface-mediated retention. For instance, bacterial cellulose membranes crosslinked with attapulgite achieved over 98% removal of PS particles ranging from 100 nm to 1 µm under batch conditions, leveraging tunable pore structure and electrostatic interactions [97]. Similarly, electrospun polyamide 6.9 (PA 6.9) membranes exhibited 99.8% removal of PS microparticles, benefiting from their highly porous nonwoven structure and hydrophobic surface, while maintaining performance over repeated reuse cycles [98]. Multi-component hydrogels based on CS, sodium alginate, and GO further illustrate the advantages of combining different functional components, achieving removal efficiencies of 97 and 99.9% for PS nanoparticles of 50 and 500 nm, respectively [99].

Synthetic polymer membranes also demonstrate strong MP retention capabilities. PES ultrafiltration membranes achieved 70–100% removal of PE microparticles in various water matrices, including wastewater, surface water, and seawater, highlighting the importance of pore architecture and surface chemistry [100]. In addition, PVDF nanofibrous membranes functionalized with quaternary ammonium groups enhanced electrostatic and hydrophobic interactions, achieving over 92% removal of PS nanoparticles in gravity-driven systems, combining size exclusion and adsorption-like retention mechanisms [45]. Further modification of PVDF nanofibers with biosurfactants and TiO_2_/CuO nanoparticles enabled up to 99.99% removal of 0.5 µm MPs in wastewater, demonstrating improved permeability and antifouling behavior [101].

Hybrid membrane systems integrating polymers with inorganic or functional additives provide additional performance improvements. Aluminosilicate media modified with cationic polymeric surfactants achieved over 96% removal of PE and PA microparticles, combining electrostatic attraction and physical entrapment [102]. Likewise, polyacrylonitrile (PAN)—reduced graphene oxide (rGO) composite membranes achieved >82% rejection of MPs in industrial wastewater while maintaining antifouling performance and reusability [46]. Additionally, palm kernel shell biochar functionalized with cetyltrimethylammonium bromide (CTAB) demonstrated >90% removal of PE MPs/NPs (159 nm–48 µm) and PA MPs (6–9 µm) through combined electrostatic attraction, hydrophobic interactions, and cake layer formation [103].

These studies demonstrate that filtration-based removal of MPs and NPs relies on a synergistic combination of size exclusion, surface interactions, and material functionalization. Natural, synthetic, and hybrid polymeric systems provide highly adjustable platforms in which pore structure, surface chemistry, and hierarchical architecture can be engineered to optimize retention efficiency, minimize fouling, and enable long-term reuse (Table 2).

### 3.3. Coagulation/Flocculation

Coagulation and flocculation (C/F) are process-driven strategies for the removal of MPs and NPs from aqueous environments. These processes rely on fundamental interaction mechanisms such as polymer bridging, charge neutralization, and electrostatic or hydrophobic interactions. In this context, FPMs act as flocculants or coagulant aids, promoting particle destabilization and aggregation into larger flocs that can subsequently be removed by sedimentation, flotation, or filtration.

Polymeric flocculants used for MP/NP removal can be broadly classified into natural, synthetic polymers, and hybrid systems combining organic and inorganic coagulants. Natural polymers are generally more sustainable and environmentally benign but may exhibit lower charge density and limited tunability. In contrast, synthetic polymers offer higher molecular control, higher charge densities, and tunable architectures, while hybrid systems combine the advantages of both approaches, often improving overall performance in complex water matrices.

The efficiency of C/F processes is strongly governed by polymer physicochemical properties, including molecular weight, charge density, and chain conformation. High-molecular-weight polymers enhance interparticle bridging by enabling adsorption onto multiple particles simultaneously, whereas highly charged polymers promote charge neutralization and destabilization of colloidal suspensions. In addition, extended-chain conformations improve surface coverage and increase the probability of particle–polymer interactions, thereby enhancing aggregation efficiency. The surface chemistry of MPs and NPs also plays a critical role in determining flocculation behavior. Pristine plastics such as PE and PP are typically hydrophobic and exhibit low surface charge, resulting in relatively stable suspensions. However, environmental aging and photo-oxidation introduce oxygen-containing functional groups, increasing hydrophilicity and negative surface charge. These changes enhance interactions with cationic polymers and significantly alter aggregation pathways.

**Figure 11:**
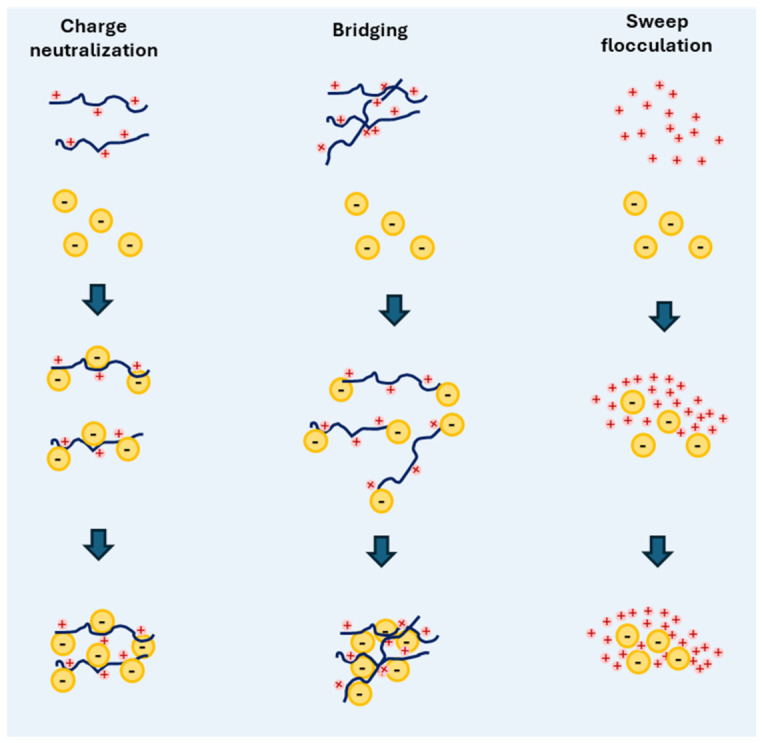
Schematic illustration of flocculation mechanisms for microplastic removal using functional polymers.

Among the governing mechanisms, polymer bridging plays a central role, whereby long-chain polymers adsorb onto multiple particles simultaneously, forming interconnected aggregates. In contrast, charge neutralization involves the adsorption of oppositely charged polymers onto particle surfaces, reducing electrostatic repulsion and enabling aggregation without the need for polymer-mediated links. Sweep flocculation, although typically associated with metal-based coagulants, occurs when particles become enmeshed within metal hydroxide precipitates formed during coagulation and may also take place in polymer-assisted systems. Hydrophobic interactions and van der Waals forces further contribute to the stabilization of the resulting flocs. Under optimized conditions, polymer-based C/F systems have been reported to achieve removal efficiencies exceeding 90% for MPs [104]. The mechanistic pathways of charge neutralization, polymer bridging, and sweep flocculation are illustrated in Figure 11.

Natural polymers have attracted considerable attention due to their biodegradability and strong affinity toward particulate matter [105]. *Opuntia Milpa Alta* (OMA) cactus mucilage combined with polymeric ferric sulfate achieved up to 93.6% removal of PS microparticles via charge neutralization, adsorption bridging, and floc entrapment, while also reducing inorganic coagulant demand [106]. When combined with polyaluminum ferric chloride (PAFC), OMA polysaccharides further enhanced coagulation performance, achieving 94.8% of PS removal through synergistic charge neutralization and bridging mechanisms [107]. Marine polysaccharides also show strong potential, as laminarin combined with polyaluminum chloride (PAC) increased PE microparticles removal from 79.2% (PAC alone) to 91.5%, highlighting synergistic interactions between polymer bridging and charge neutralization [108].

In addition to plant- and marine-derived materials, microalgae, fungi, and their EPS provide environmentally friendly flocculation routes. A system combining *Chlorella vulgaris* and *Aspergillus niger* pellets promoted hetero-aggregation of PP and PET microparticles. The removal of 55.7% (PP) and 95% (PET) reached by *Chlorella* was further increased to 90% and 94% upon the addition of fungal bioflocculant [63]. EPS produced by algae and bacteria can either stabilize or destabilize colloids depending on ionic strength and surface chemistry. For example, increasing NaCl concentration (200–1400 mM) generally enhanced steric stabilization, whereas CaCl_2_ (10–100 mM) could either promote or inhibit aggregation depending on functional groups. Reported critical coagulation concentrations in the presence of EPS (4 mg C/L) were 70 mM for NaCl and 1.5 mM for CaCl_2_, illustrating the strong influence of ionic composition on aggregation behavior [109].

**Figure 12:**
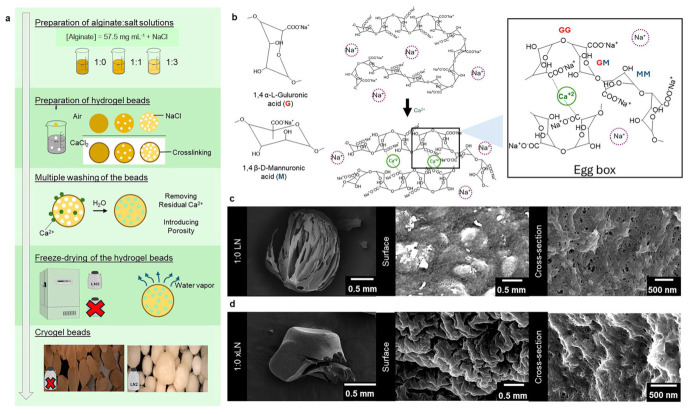
Preparation of alginate cryogel beds. (**a**) Experimental process to fabricate alginate cryogel beads. (**b**) Alginate chemical structure, where the purple dashed circles represent Sodium ions, and the green solid circles represent the Calcium ions. (**c**) Freeze-dried following liquid nitrogen treatment (Alg LN 1:0), with SEM images taken at magnifications of 37, 33.75 K, and 30 K from left to right, respectively. (**d**) Freeze-dried without liquid nitrogen treatment (Alg xLN 1:0), with SEM images taken at magnifications of 53, 10 K, and 30 K from left to right, respectively. Without liquid nitrogen treatment, the alginate surface shrank into a wrinkled morphology, increasing surface topology and reducing effective pore size. In (**c**,**d**), each row presents the whole view, surface, and cross-section of alginate beads. Scale bars are given in each image. Reprinted from reference [35].

**Figure 13:**
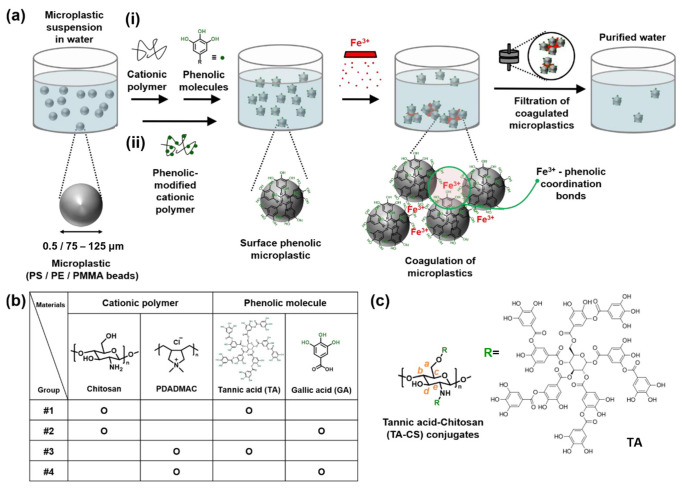
(**a**) Schematic illustration of microplastic (MP; 0.5/75–125 µm) removal in water. Polystyrene (PS), polyethylene (PE), and poly(methyl methacrylate) (PMMA) beads were used to develop the MP model. The MP surface was treated with (i) a cationic polymer (chitosan or poly(diallyldimethylammonium chloride) (PDADMAC)) and phenolic molecule (tannic acid or gallic acid) or (ii) phenolic-modified cationic polymer to form surface phenolic MPs. Next, Fe^3+^ triggered the coagulation of the MPs via Fe^3+^-phenolic coordinate bonds. The coagulated MPs were filtered for removal, resulting in purified water. (**b**) Experimental groups of combined cationic polymer and phenolic molecule used for MP surface treatment. (**c**) Structure of tannic acid–chitosan (TA–CS) conjugates as a phenolic-modified cationic polymer to function as a single coagulant. Reprinted from reference [114].

Cationic polymers such as CS have also demonstrated high efficiency in both MP and NP removal. CS-modified air flotation of PS nanoparticles (~100 nm) increased removal efficiency from 3.1% (conventional flotation) to 96.7%, maintaining 92.8% under reduced flotation times. Mechanistic analyses indicate that CS enhances aggregation via electrostatic attraction, polymer bridging, and hydrophobic interactions with air bubbles, supported by zeta potential and contact angle measurements [110]. CS and sodium alginate systems further improve floc formation and settling behavior for PS, PE, and PET microparticles in wastewater treatment, while reducing coagulant demand [111]. In contrast, calcium alginate hydrogels have shown removal efficiencies exceeding 99% for PS microparticles through effective particle enmeshment within polymeric flocs [112]. Alginate cryogels have achieved over 99% removal of PS and PE nanoparticles by rapidly inducing particle aggregation through alginate adsorption and Ca^2+^-mediated charge destabilization, effectively incorporating NPs into polymer networks for subsequent removal [35]. Figure 12 illustrates the preparation of alginate cryogel beds.Synthetic polymeric flocculants provide additional tunability through controlled chemical design. Cationic polyacrylamide (PAM) combined with PAC or polyferric sulfate (PFS) significantly enhanced PS microparticles removal, achieving 87.5% (PAC/cationic PAM) and 62.5% (PFS/cationic PAM), outperforming anionic and nonionic counterparts and maintaining performance across multiple plastic types (PE, PP, PET, PVC) [113]. Chemically modified polymers such as tannic acid-CS conjugates act as cationic phenolic flocculants, inducing aggregation via metal-phenolic coordination in the presence of Fe^3+^ ions, achieving 78–89% PS removal within 5 min, compared to 52–54% using tannic acid-CS alone [114]. Figure 13 illustrates the coagulation process using a combination of cationic polymers and phenolic molecules.

Hybrid coagulation systems combining inorganic coagulants with functional polymers further enhance removal efficiency. For example, Al_2_(SO_4_)_3_ combined with *Moringa Oleifera* (MO) or anionic PAM achieved 93–94% removal of PA, 80–81% of PS, and 28–29% of PE microparticles. Mechanistic analysis indicated combined charge neutralization and polymer bridging, while reducing alum dosage by up to 50% in MO-assisted systems [115].

Table 3 summarizes the reported removal efficiencies of MPs and NPs using polymeric coagulation and flocculation systems.

## 4. Comparative Performance and Key Challenges

The diverse functional polymeric materials (FPMs) discussed in previous sections exhibit a wide range of efficiencies, mechanisms, and operational behaviors for the removal of micro- and nanoplastics from aqueous systems. However, direct comparison between material classes remains challenging due to variability in particle type, size distribution, environmental matrix, and experimental configuration, which complicates standardized benchmarking of adsorption, filtration, or coagulation performance. Addressing these inconsistencies requires the development of harmonized testing protocols and representative evaluation conditions.

Several key challenges are consistently reported across FPM systems, including fouling, long-term stability, material regeneration, and scalability. In particular, fouling can be further differentiated into cake layer formation, pore blockage, and biofilm development, each of which affects permeability, flux decline, and operational continuity in distinct ways. These limitations are particularly relevant for membrane- and filtration-based systems, where the accumulation of organic matter, biofilms, and plastic particles can reduce operational efficiency over time. While surface functionalization and antifouling strategies can mitigate these effects, sustained performance under complex water matrices remains insufficiently demonstrated.

Hybrid and multifunctional materials offer enhanced versatility by integrating multiple removal mechanisms, enabling the capture of heterogeneous MPs/NPs. However, their increased structural and chemical complexity is often associated with higher synthesis costs and potential limitations in large-scale implementation.

A detailed and systematic critical evaluation of FPM classes, including their specific advantages, limitations, and research gaps, is provided in the Critical Evaluation and Research Gaps Section.

### Critical Evaluation and Research Gaps

Despite the broad range of functional polymeric materials (FPMs) reported for MPs and NPs removal, a critical comparison across material classes reveals distinct advantages, limitations, and unresolved challenges that are not always explicitly addressed in the literature.

Natural and polysaccharide-derived materials generally exhibit high adsorption capacities, particularly for NPs, due to their rich functional group density and tunable three-dimensional networks. However, their practical use is often limited by structural instability, swelling behavior, and partial degradation during repeated adsorption–desorption cycles, which reduces long-term reusability. In contrast, synthetic polymer composites and membrane-based systems provide higher mechanical robustness and more stable performance over multiple cycles but are more prone to fouling phenomena such as pore blockage, cake formation, and biofilm accumulation, especially under realistic water conditions.

Hybrid and multifunctional materials, including magnetic composites and MOF–polymer systems, combine multiple interaction mechanisms and enable efficient recovery and multi-target capture. Nevertheless, their complex synthesis routes, higher cost, and potential scalability limitations remain key barriers for practical deployment.

From a mechanistic perspective, MP removal is mainly governed by size exclusion and physical entrapment, whereas NP capture relies more strongly on electrostatic interactions, hydrogen bonding, hydrophobic effects, and π–π stacking. These interactions are highly sensitive to environmental parameters such as pH, ionic strength, temperature, and NOM, which can either enhance aggregation or reduce adsorption efficiency through competitive or screening effects. Despite this, relatively few studies systematically evaluate performance under environmentally relevant conditions, limiting the transferability of laboratory results.

Fouling remains a major limitation in membrane- and filtration-based systems and occurs through multiple mechanisms, including cake layer formation on the membrane surface and pore blockage within the membrane structure. While cake formation may, in some cases, enhance MP retention by acting as a secondary filtration layer, pore blockage significantly reduces membrane permeability and long-term operational stability. In addition, NOM and colloidal species can exacerbate fouling by forming compact and heterogeneous layers that hinder water flux.

To address these challenges, various mitigation strategies have been explored, including hydrophilic and antifouling coatings, surface functionalization with charged or zwitterionic groups, and the incorporation of nanomaterials to reduce foulant adhesion and improve permeability. Despite these advances, maintaining long-term performance under realistic environmental conditions remains a critical challenge.

A further limitation lies in the lack of standardized experimental protocols. Differences in particle type, size distribution, concentration, and water matrix composition hinder direct comparison between studies and prevent robust benchmarking of FPM performance. In addition, most reported studies are conducted under simplified laboratory conditions, with limited consideration of complex real-water matrices.

Finally, sustainability and economic considerations remain insufficiently integrated into material development. Natural and polysaccharide-based polymers offer clear advantages in terms of renewability, biodegradability, and low environmental impact; however, their structural instability and limited durability under repeated use reduce their long-term applicability. In contrast, synthetic polymers and composite systems provide improved mechanical strength and operational stability but raise concerns related to environmental footprint, resource intensity, and end-of-life disposal. Recent studies have addressed these limitations through the development of recyclable polymer systems and the incorporation of natural or partially biodegradable components into hybrid materials. Only a limited number of studies include life-cycle or techno-economic assessments, which are essential for evaluating real-world applicability.

Overall, current research tends to prioritize removal efficiency under controlled conditions, whereas long-term stability, regeneration, environmental compatibility, and scalability remain underexplored. Addressing these gaps will require standardized testing frameworks, mechanistic studies under realistic conditions, and integration.

## 5. Conclusions and Future Perspectives

Functional polymeric materials (FPMs) represent a versatile platform for the removal of MPs/NPs from aqueous systems due to their tunable chemical composition, structural diversity, and ability to promote multiple interaction mechanisms. A wide range of systems, including hydrogels, aerogels, membranes, nanocomposites, natural polymers, and hybrid materials, has demonstrated promising removal efficiencies under laboratory conditions.

Removal performance is governed by the interplay between polymer functional groups, material morphology, and the physicochemical properties of plastic particles. Hydrophobic domains, charged functional groups, and aromatic structures enable adsorption, electrostatic attraction, hydrogen bonding, and π–π interactions, while pore architecture and network design control diffusion and physical entrapment. In this context, MP removal is often dominated by size exclusion and physical capture, whereas NP removal typically requires stronger interfacial interactions and higher surface area or functionalization.

Recent advances in hybrid and multifunctional materials have further improved removal efficiency and reusability. Magnetic systems enable facile separation and recycling, while MOF-polymer composites offer high surface area combined with tunable chemistry. Natural polymers provide environmentally compatible alternatives, although trade-offs in mechanical stability and long-term durability may arise. These developments highlight the potential of FPMs to address both controlled laboratory systems and more complex real-world water treatment scenarios.

Despite these advances, significant challenges remain for practical implementation, particularly regarding standardization, scalability, and long-term performance under environmentally relevant conditions. Additional limitations include fouling, material regeneration, and the lack of systematic evaluation in complex water matrices. Furthermore, the heterogeneous nature of environmental plastics, including aging effects and additive content, adds complexity to the design of selective removal systems.

Sustainability considerations are increasingly important in material development. Natural polymers offer advantages in terms of renewability and low environmental impact, whereas synthetic systems provide superior mechanical stability and tunable performance but raise concerns related to environmental footprint and end-of-life management. Hybrid approaches combining both material classes, along with recyclable and low-impact design strategies, represent promising pathways to balance performance and sustainability.

Future research should focus on standardized testing protocols, mechanistic studies under realistic environmental conditions, and the development of robust structure-property design criteria to enable targeted interactions with heterogeneous MP/NP mixtures. In addition, integrating multifunctional properties, such as antifouling behavior, mechanical robustness, and regeneration capacity, will be essential to enable scale-up and long-term operation.

Overall, the successful implementation of functional polymeric materials will depend on a shift toward application-driven design strategies, in which material development is guided not only by removal efficiency but also by long-term stability, reusability, sustainability, and system-level integration. This holistic approach will be essential to bridge the gap between laboratory research and practical water treatment applications.

## Figures and Tables

**Table 1:** Summary of adsorption-based removal of micro- and nanoplastics by functional polymeric materials.

Adsorbent Material	Target MP/NP	Capacity (mg·g^−1^)/Efficiency (%)	Conditions (pH)	Reuse (Cycles)	Mechanism/Features	Ref.
Chitin–cationic lignin composite hydrogel	PS (166 nm)	1790.8 mg·g^−1^, 93.7%	pH 7 batch	>3 cycles	Porous, cationic; electrostatic interactions, π–π stacking	[32]
PDA-modified sodium alginate hydrogel	PS-NH_2_ (200 nm) PS-COOH (300 nm)	127.98–154.57 mg·g^−1^ ~99.6%	pH 7–8 batch	-	Elastic, thermally stable polymer; chemical adsorption, electrostatic interactions, H-bonding, π–π stacking	[30]
PNIPAM and sodium carboxymethyl cellulose hydrogel	PS-NH_2_ (<100 nm)	199.64 mg·g^−1^ 99.83%	pH 6.28 batch	5 cycles	High adsorption efficiency in multi-ionic systems; chemical adsorption, electrostatic interactions, H-bonding, π–π stacking, pore filling	[71]
Polysaccharide aerogels (CS–CNF–PDA)	PS-COOH, PS, PMMA, PE, PP (500 nm)	>300 mg·g^−1^, >96%	pH 6–8 batch and continuous flow	4 cycles	Hydrophobic porous structure, bidirectional porosity; electrostatic interactions, H-bonding, van der Waals, π–π stacking, hydrophobic interactions	[72]
GO–nanocellulose aerogel	PS (1 µm)	241.6 mg·g^−1^ 91.6%	pH 7 batch	20 cycles	Bidirectional layered structure, high surface area; π–π stacking	[73]
Quaternary ammonium-modified cellulose nanofiber aerogel	PS (1 µm)	146.3 mg·g^−1^ -	pH 7 batch	2 cycles	Directional pores, cationic surface; chemisorption, electrostatic interactions, physical entrapment	[74]
Cellulose nanofiber-/PEI-modified aerogel	PS (1 µm)	117 mg·g^−1^ -	pH 7 batch	1–2 cycles	Directional structure; chemisorption, electrostatic interactions	[75]
Trifunctional anthracene and bis-triazolinedione mesoporous polymer	PS-COOH (1 µm), PS (>500 nm)	190 mg·g^−1^ 99%	pH 6 batch and continuous flow	5 cycles	Multifunctional mesoporous polymer, strong chemical resistance, hydrophobic; π–π stacking, H-bonding, hydrophobic interactions	[78]
Chitin–GO porous sponge	neat PS, PS-COOH, PS-NH2	5.9–8.4 mg·g^−1^ 72.4–89.8%	pH 6–8 batch	3 cycles	Good elasticity, high porosity; electrostatic interactions, H-bonding, π–π stacking	[79]
Chitin-based sponges with GO and oxygen-doped carbon nitride (O-C3N4)	PS-COOH, PS-NH2, PS (1 µm)	3.93–8.79 mg·g^−1^ -	pH 6–8 batch	3 cycles	High mechanical strength, excellent elasticity; electrostatic interactions, H-bonding, π–π stacking	[51]
Cellulose–MgAl–LDH composite	PS (100 nm)	6.08 mg·g^−1^ 90%	pH 2–7 batch	2 cycles-	Composite beds (4 mm); intra-particle diffusion, H-bonding, electrostatic interactions	[57]
Graphene-like carbon-assembled layered double oxide from organic LDH	PS (100 µm)	- 90%	pH 7–11 batch	5 cycles	3D graphene-like carbon/layered double oxide; H-bonding, π–π/p-π interactions	[80]
Magnetic iron-modified biochar composite	PS-COOH (30 nm and 1000 nm), PS-NH2 1000 nm)	206–290 mg·g^−1^ 82–95%	pH 3–10 batch	4 Cycles	Magnetic composite, spherical iron oxide nanoparticles on biochar; complexation, electrostatic interactions	[81]
Magnetic Fe_3_O_4_-Biochar	PS-COOH, PS-NH2, PS (100 nm)	107–229 mg·g^−1^ 47.7–95.2%	pH 3–7 batch	-	Magnetite coating, ultrafine composite; electrostatic interactions, H-bonding, π–π stacking	[82]
Iron-modified, magnetic char from discarded cigarette butts	PS (1 µm)	- 87.6% (deionized water) 54–79% (real waters)	pH 4–8	5 cycles	Magnetic recycled adsorbent; hydrophobic interactions, π–π stacking, pore filling, electrostatic interactions	[83]
Fe_3_O_4_/PSS/ZIF-67 magnetic composite	PS, PP, PE, PET, PMMA, PVC, polyamide (PA)	2420–2897 mg·g^−1^ 93%	pH 1.4–13.1	3 cycles	Multifunctional magnetic adsorbent; electrostatic interactions, π–π stacking, H-bonding, van der Waals	[84]
ZIF-8/Wood aerogel composite	PVDF (60–110 nm), PS (90–140 nm)	- >85–91%	-	3 cycles	MOF grown on aerogel, improved stability; electrostatic interactions, H-bonding, hydrophobic interactions, van der Waals	[85]
ZIF-8/PDA/Diatomite	PS 100 nm	- 91.53%	pH 6–7 batch	7 cycles	MOF + polymer coating, multifunctional; electrostatic interactions, H-bonding, π–π stacking	[86]
ZIF-8/sodium alginate/PDMS	PMMA (5 µm), PVDF (200 nm), and polyvinyl chloride (PVC) (1 µm)	282–594 mg·g^−1^ >80%	pH 7 batch	7 cycles	MOF grown on polymer framework, monolithic hydrophobic adsorbent; electrostatic interactions, H-bonding, hydrophobic interactions, van der Waals	[41]
UiO-66-NH_2_ mesoporous MOF	PS 26 nm	524 mg·g^−1^ 100%	pH 7 batch	-	Zirconium-based, mesoporous, functionalized; electrostatic interactions, van der Waals	[87]

**Table 2:** Summary of filtration-based removal of micro- and nanoplastics by functional polymeric materials.

Filter/Membrane Material	Pore Size (nm)	Target MP/NP	Removal Efficiency	Conditions (pH)	Reuse (Cycles)	Mechanism/Features	Ref.
Bacterial cellulose + attapulgite	14.87–339.9	PS 100 nm–1 µm	>98%	pH 1–14	10 cycles	Compact microporous structure; size exclusion, pore trapping, electrostatic repulsion	[97]
PA 6.9 electrospun membrane	550–1140	PS 679 nm	99.8%	-	10 cycles	Hydrophobic, highly porous nonwoven, high surface roughness; surface interaction, size exclusion, cake formation	[98]
Sodium alginate/CS-modified GO	-	PS 50 nm, 500 nm	>97%	pH 1–13	10 cycles	Multilayer SA/GO/CS composite membrane; size exclusion, electrostatic repulsion + electrostatic adsorption	[99]
PES UF membrane	20	PE 10–150 µm	70–100%	-	-	Ultrafiltration membrane, high porosity, stable polymer; size exclusion	[100]
PVDF nanofibers + quaternary ammonium	-	PS 107–1450 nm,	>92%	pH 6.8–11.2	10 cycles	Cationic nanofibrous membrane; size exclusion, electrostatic attraction, hydrophobic interactions	[45]
PVDF + biosurfactant + TiO_2_/CuO	540–740	PS 0.5 µm	99.99%	-	-	Enhanced permeability, antifouling, surface roughness; electrostatic attraction, surface adsorption	[101]
Aluminosilicate + cationic polymeric surfactant	-	PE 10 µm PA 100 µm	>96%	-	-	Aluminosilicate filter media, cationic surfactant-modified, hydrophobic; electrostatic bonding, captured/trapped/entangled retention	[102]
PAN/reduced graphene oxide (rGO) composite	153–203	PET <150 nm	>82%	-	-	Composite membrane, tunable porosity, anti-fouling; size exclusion, surface interaction	[46]
Palm kernel shell biochar + CTAB	993–1190	PE 159 nm–48 µm PA 6–9 µm	>95%	pH 7		Cationic surfactant, positive surface charge, hydrophobic; electrostatic attraction, hydrophobic interactions, physical retention (cake layer formation)	[103]

**Table 3:** Summary of coagulation/flocculation-based removal of micro- and nanoplastics by functional polymeric materials.

Coagulant/Flocculant Material	Target MP/NP	Capacity (mg·g^−1^)/Efficiency (%)	Conditions (pH)	Mechanism/Features	Ref.
*Opuntia Milpa Alta* mucilage + polymeric ferric sulfate	PS (2–10 μm)	93.6%	pH 9.2 Jar test, batch	Cactus polysaccharides, mucilage-functional groups; charge neutralization, adsorption bridging, floc entrapment	[106]
*Opuntia Milpa Alta* + PAFC	PS (2–10 μm)	94.8%	pH 9 Jar test, batch	PAFC-OMA composite, rough surface, mesh structure; charge neutralization, adsorption bridging	[107]
Laminarin + PAC	PE (50–150 μm)	91.5%	pH 8 batch	PAC-Laminarin composite, polysaccharide functional groups; charge neutralization, sweep flocculation, and adsorption bridging	[108]
*Chlorella vulgaris* + *Aspergillus niger* EPS	PP, PET	90% (PP) 95% (PET)	batch	Microalgae + fungal EPS, EPS-mediated bridging, hetero-aggregation, enhanced MP binding	[63]
FeCl_3_-CS/FeCl_3_-Sodium alginate composite	PS, PE, PET (<500 µm)	PS: 93.77% (FeCl_3_-SA), PE: 97.81% (FeCl_3_-CT), PET: 98.39% (FeCl_3_-SA)	pH 7 Jar test, batch	Natural polymeric coagulant aid; sweep flocculation, polymer bridging	[111]
Calcium alginate hydrogel	PS, PE, PP, PA, PMMA, PLA (5–150 µm)	52–99.5%	pH 6–9 batch	Crosslinked polysaccharide; encapsulation, sweep/floc entrapment, minimal electrostatic effect	[112]
Alginate cryogel	PS, PE (50–200 nm)	>99% for PS and PE NPs (50–200 nm)	pH 4–8	Alginate cryogel, Ca^2+^-mediated aggregation; adsorption, charge destabilization, aggregation, enmeshment	[35]
PAC + PAM	PE, PP, PET, PVC MPs	63.75–87.5% (PS)	pH 7 batch	Cationic synthetic polymer, enhanced coagulation, multi-polymer removal; charge neutralization, polymer bridging, electrostatic bridging	[113]
Tanic acid-CS + Fe^3+^	PS (90 µm) PE (106–125 µm) PMMA (75–90 µm)	78–89%	- batch	Phenolic-cationic polymer, metal coordination, Fe^3+^-assisted coagulation; metal-phenolic coordination, polymer bridging, electrostatic attraction	[114]
Al_2_(SO_4_)_3_ + *Moringa oleifera*	PA, PS, PE (<500 µm)	93% (PA) 80% (PS) 29% (PE)	pH 9 Jar test, batch	Hybrid coagulation system, reduced coagulant dosage; charge neutralization, polymer bridging, adsorption	[115]

## Data Availability

No new data were created or analyzed in this study. Data sharing is not applicable to this article.

## Abbreviations

The following abbreviations are used in this manuscript:

AFMs	Advanced functional membranes
C/F	Coagulation/flocculation
CNTs	Carbon nanotubes
CS	Chitosan
CTAB	Cetyltrimethylammonium bromide
EPS	Extracellular polymeric substances
GO	Graphene oxide
LDH	Layered double hydroxides
MWCO	Molecular weight cut-off
MF	Microfiltration
MOFs	Metal-organic frameworks
NaCMC	Sodium carboxymethyl cellulose
NF	Nanofiltration
NOM	Natural organic matter
OMA	Opuntia Milpa Alta
PA	Polyamide
PAC	Polyaluminum chloride
PAFC	Polyaluminum ferric chloride
PAM	Polyacrylamide
PAN	Polyacrylonitrile
PDA	Polydopamine
PDMS	Polydimethylsiloxane
PE	Polyethylene
PEI	Polyethyleneimine
PES	Polyethersulfone
PET	Polyethylene terephthalate
PFS	Polyferric sulfate
PMMA	Poly(methyl methacrylate)
PNIPAM	Poly(N-isopropylacrylamide)
PP	Polypropylene
PS	Polystyrene
PS–COOH	Carboxylated polystyrene
PS–NH_2_	Amino-functionalized polystyrene
PSF	Polysulfone
PSS	Poly(sodium p-styrenesulfonate)
PVC	Poly(vinyl chloride)
PVDF	Poly(vinylidene fluoride)
RO	Reverse osmosis
UF	Ultrafiltration
ZIF	Zeolitic imidazolate framework
